# The tuning of the plasmon resonance of the metal nanoparticles in terms of the SERS effect

**DOI:** 10.1007/s00396-018-4308-9

**Published:** 2018-04-16

**Authors:** Z. Starowicz, R. Wojnarowska-Nowak, P. Ozga, E. M. Sheregii

**Affiliations:** 1 Institute of Metallurgy and Materials Science, Polish Academy of Sciences, 25 Reymonta Str, 30-059 Krakow, Poland; 2 Centre for Microelectronics and Nanotechnology, University of Rzeszow, 1 Pigonia Str, 35-959 Rzeszow, Poland

**Keywords:** Plasmonics, Metal nanoparticles, Colloids, SERS, Core-shell, Bionanocomplexes

## Abstract

The Surface-enhanced Raman spectroscopy is the essential tool for various levels of the molecular studies. In order to become widely used as a fast analytical tool, the enhancing structures such as the nanoparticles have to be simple, inexpensive, and offer good flexibility in enhancing properties and the spectral range. In this paper, we investigated the plasmonic properties of the metal nanoparticles, to which the molecules of interest can be adsorbed, forming the bionanocomplexes. Here, for the first time, we provided the collection of the results gathered in one article, which can serve as the basis or guidance for designing the SERS studies on different bionanocomplexes, various nanoparticle structures, sizes, and excitation wavelengths. The presented plasmonic properties describe the spectral position of the plasmonic resonances as results of their size and structure. The electric field enhancement as a key contributor to the SERS effect is given as well. We considered silver and gold nanoparticles and their variations. Gold is one of the best choice, due to its relevant surface properties, however, suffers from the plasmonic activity and rather static spectral position of the plasmonic resonances. Therefore, one of the main purposes was to show the effective resonance tuning using simple and less expensive geometries. We showed the possibility to adjust the plasmonic resonances with the excitation wavelengths from the blue region to the near infrared region of lasers most commonly used for Raman spectroscopy. The presented studies indicated the high potential of the core-shell structures for this kind of applications.

## Introduction

The analysis of the oscillation spectra of the biological molecules can give the information about the chemical structure, as well as interactions between the molecules [1, 2]. The Infrared and Raman spectroscopy techniques are successfully used for the study of the vibrational fingerprints of the biomolecules. In addition, the recorded spectra reveal the information about the presence of the chemical bonds and the functional groups, as well as the secondary structure of the proteins. This makes the oscillation spectra the valuable tools for the investigation of the protein structure [1, 3]; of the molecular mechanism of the proteins reactions [4]; and of the protein folding, unfolding, and misfolding [5]. Raman spectroscopy is the method of testing the vibrational spectra of various substances including the proteins [3, 6]. This technique has a great potential for obtaining information from a wide range of materials in biology and medicine, and especially for the purpose of non-invasive control and diagnostics [6]. Raman scattering is a weak phenomenon (normally only one photon from 10^6^ to 10^8^ scattered photons takes part), therefore surface-enhanced Raman spectroscopy (SERS) technique is more promising. SERS is mainly based on the phenomenon of the largely increased Raman scattering, accomplished by placing the investigated molecules on the surface of the metallic nanostructures such as the nanoparticles (NPs) or the rough surfaces. The combination of the high sensitivity and selectivity makes it an advantageous analytical tool in broad applications both in the biological detection, as well as, in the medical diagnostics [7–9]. Since its discovery in 1974 [10], the SERS technique has been strongly developed. The signal enhancement factors reaches up to 10^15^ [11] and allows the detection of even single molecules [7]. The two basic mechanisms are responsible for the increase of the Raman signal. Firstly, it is connected with the increase of Raman scattering cross section of the molecule, due to the modified environment in the vicinity of the molecule and also with the chemisorption of the molecules on the metal surface (the new chemical bond, the molecule-metal charge transfer mechanism). It is called the chemical contribution to the signal enhancement; however, it can introduce changes to the order of 10^2^. Much higher enhancement is possible, due to the second so called electromagnetic contribution. Thanks to the lightning rod effect on the surface roughness and plasmonic excitations, mostly the Localized Surface Plasmons (LSPR), the electric field can be locally drastically enhanced. Due to the Raman shift being small, compared to the LSPR bandwidth, both incoming and emitted light fields are enhanced. Therefore, the electromagnetic enhancement factor corresponds approximately with the field enhancement to the power of four (E_loc_/E_0_)^4^. It shows the crucial requirement to match the excitation light wavelength and the SERS geometry in order to take advantage from the resonantly enhanced fields [12]. Additionally, the observation of the large Raman signals, the placement of the investigated molecules in close proximity of the metal nanoparticles, or the metal-nano-structured surface is the necessary condition. Moreover, the number of factors have the significant impact on the achieved results: the type of noble metal used as the SERS substrate (with its optical properties) [13], shape and size of the resonant nanostructures [14, 15]. The presence of the aggregates and the interparticle space is also important [16]. The obtained Raman spectra strongly depend on the laser excitation wavelength and it should be adjusted to the absorption range of the studied molecules [6]. In case of the SERS, the wavelength and the polarization of the excitation light is even more important [17, 18].

The biomolecules (the enzymes or the antibodies, for example) bounded with the metal nanoparticles can be useful in the modern diagnostic methods to develop the sensitive optical biosensors [19]. Therefore, in the presented paper, the plasmonic properties of the metal nanoparticles that can significantly contribute to the SERS enhancement were considered. It is known that any variation in the structure and the shape of the SERS nano-carriers alters the plasmonic activity and their spectral position. Therefore, all of the parameters should be properly chosen during the designing process of the new SERS devices. The considered system of the plasmonic nanoparticles to which the investigated molecules can be attached is dedicated to the water environment. By using various developed methods of nanoparticle synthesis, we can basically create a number of particles, size, and shapes from the nanoparticles of a few to several hundred nanometers, from the simple spherical particles to the very complex nanogroups. In addition, we can modify them for a variety of the possibilities by attaching the substituents and forming the bionanocomplexes. Due to the single use, only of such nanoparticles for detecting purposes, we focused on the technologically non-complicated and cost effective geometries. Predictions of their plasmonic properties and the resonance tunability were the main purpose of this article, rather than the ultimate performances achievable in the advanced concepts. The presented theoretical and computational analysis seems to be helpful because the experimental testing of every available solution may be too durable and expensive.

## Method

The silver and the gold nanoparticles were selected for carrying out the theoretical considerations, as the most often used plasmonic materials with high enhancement factor, good air stability, and weak reactivity [13]. In the case of the SERS biosensors for the biological and the medical applications, it is important to use a neutral material, which does not change the properties of the biological molecules, particularly the catalytic properties of enzymes. Therefore, gold being more stable and unreactive seems to be more appropriate for this purpose. Furthermore, the stability of the enzymatic bioactivity in various environmental conditions and over the period of time can be improved by the presence of the gold nanoparticles in the synthesized bionanocomplexes [20]. However, silver often offers higher enhancement value and it is less cost consuming.

The simulations were performed using finite time difference domain method in commercial FDTD Solution simulation software from Lumerical. The investigated geometry was a single nanoparticle approximated by a perfect sphere suspended in the homogeneous medium, which for this case is water, with constant refractive index of 1.33. Through the applied perfectly matched boundary conditions, any interparticle interactions were ignored. The type of boundary conditions was chosen on the basis of the preliminary simulations. The scattering and the absorption cross sections were investigated by the Total Field Scattered Field (TFSF) technique, where the particle is surrounded by the TFSF source monitor analysis group. The absorption cross section was calculated from an analysis group located inside the TFSF source. Similarly, the scattering cross section was calculated from an analysis group located outside the TFSF source. The calculation of the cross section was based on the integration of the Poynting vector over the respective box surrounding the NP. The size of the box was chosen to minimize simulation time, but sufficient enough not to influence the results. The data for the absorption and the scattering cross section were taken in the range of 300–900 nm with the 2–5-nm spacing. Extinction cross section was obtained by adding the absorption and the scattering cross sections. The normalized extinction cross section, often called the extinction efficiency, was calculated after the division of the simulated extinction by the geometrical cross section of the investigated nanoparticle. The electric field distribution was obtained from the field profile monitor for the particular wavelength of interest, corresponding with the most commonly used excitation lasers in the Raman measuring equipment (*λ*_exc_) 488, 514, 633, 780, and 830 nm. The field is normalized by the E_0_ from the incoming light. The mathematically applied square of the E field enhancement was chosen for the analysis, due to the convenience. It should be remembered that the SERS electromagnetic factor is E_enh_^4^. On the electric field enhancement plots, we used the highest expected values for pixel at the verge of NP, but it should be emphasized that it does not necessary correspond directly with the SERS factor. We used the Palik [21] reference for the silver and the silicon dioxide and the Johnson & Christy [22] for gold. The refractive index of the titanium dioxide is taken from the elipsometric measurements of the sol gel titania thin films in anatase phase [23]. The main variable parameter was the size of the investigated particles considered in the geometries of the single metal sphere, the core-shell structure, and the nanorod. The other conditions of the simulations were adjusted by the preliminary test. All simulation results presented here enable the comparison of the nanoparticles properties and refer to the case of the suspensions with the constant number of NPs [24].

## Results and discussion

**Fig. 1:**
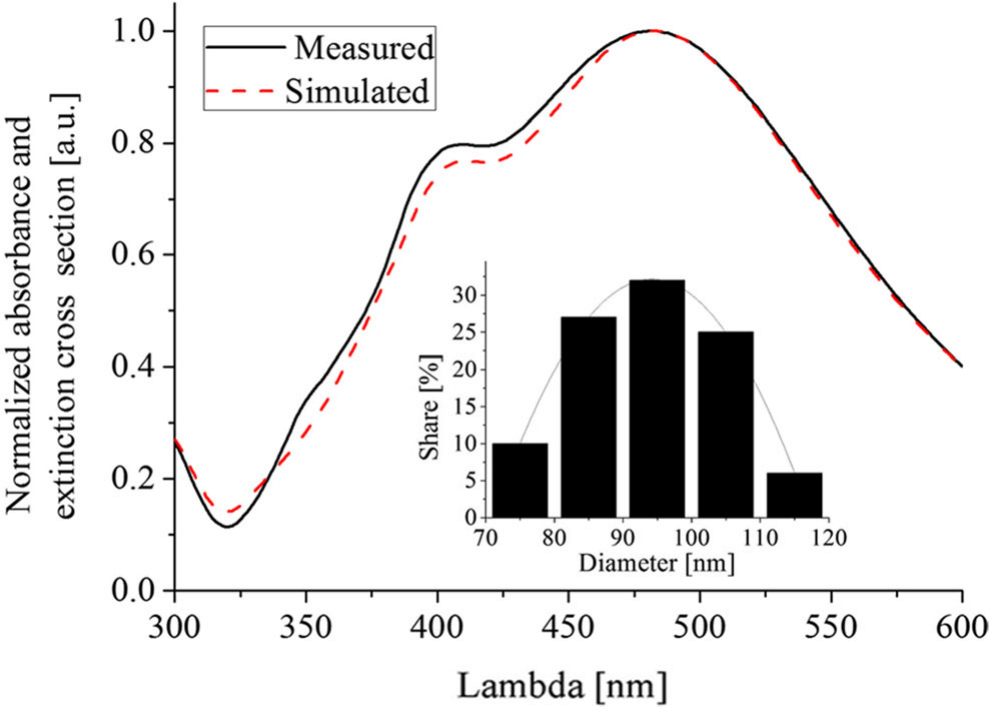
The verification of the preliminary simulation conditions using the measured absorbance of the Ag colloidal suspension and the simulated extinction cross section

The extent to which this assumption and the condition of the simulation is reasonable can be found from the comparison of the simulated extinction cross section to the absorbance of the silver nanoparticles colloidal suspension measured by the UV-Vis spectroscopy. For this purpose, the commercial Ag suspension from the NanoComposix (product name: 100 Silver Nanospheres Xact) was considered including the size of the distribution, declared be the supplier, on the basis of the TEM observation. The measured and the simulated values were normalized to unity. We obtained good agreement, which confirmed the reliability of the simulation prediction being reasonably accurate. The obtained results are shown in Fig. 1.

**Fig. 2:**
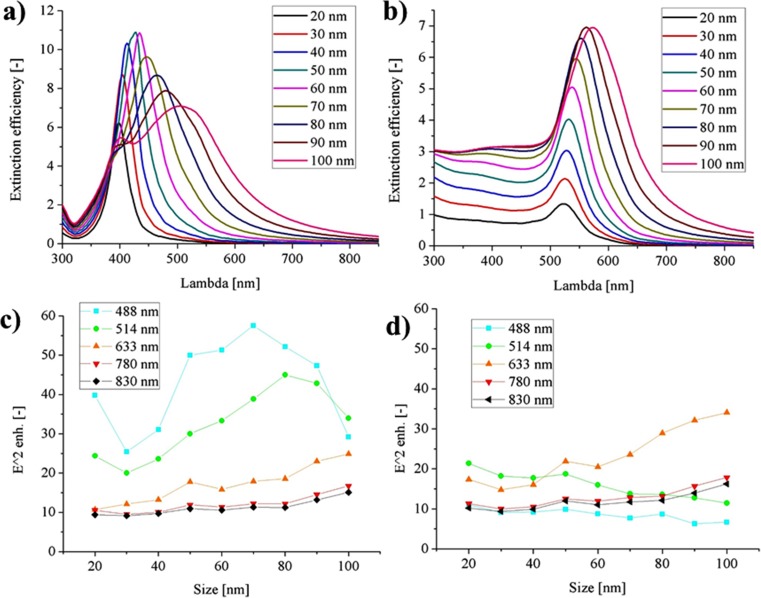
The normalized extinction cross section of the **a** silver and **b** gold spherical nanoparticles in water for the size of NPs in the range of 20–100 nm, the maximum possible E^2^ enhancement at specific locations on the NP surface, as a function of the size and the excitation wavelength **c** silver and **d** gold. The line on the E^2^ enhancement plots were added as the guide for the eye

As mentioned in the Method section, the most obvious and common choice of the plasmonic materials is silver and gold. The hence spherical single gold (AuNP) and silver (AgNP) nanoparticles were investigated at first. Figure 2 shows the extinction of the efficiency of both metals. The extinction cross sections are the measurement of how the light energy (the electromagnetic wave energy) is intercepted by the interaction with the individual plasmonic nanoparticle. It shows the position of the resonant interaction and its strength. The extinction normalized by the geometric cross section provided information on the energy concentration properties. For silver, the highest plasmonic activity is located between the 400 and 520 nm (position of the peak maxima), while for gold, it is between 525 and 580 nm. It can be seen that AuNP has a much weaker resonance shift, due to the increased nanoparticle diameter; therefore, the gold is known to have a quite static spectral resonance position. When comparing the extinction efficiency at the peak, for AgNP, it may be possible to reach the values over 10, while for AuNP even theoretically hardly reaches 7.

**Fig. 3:**
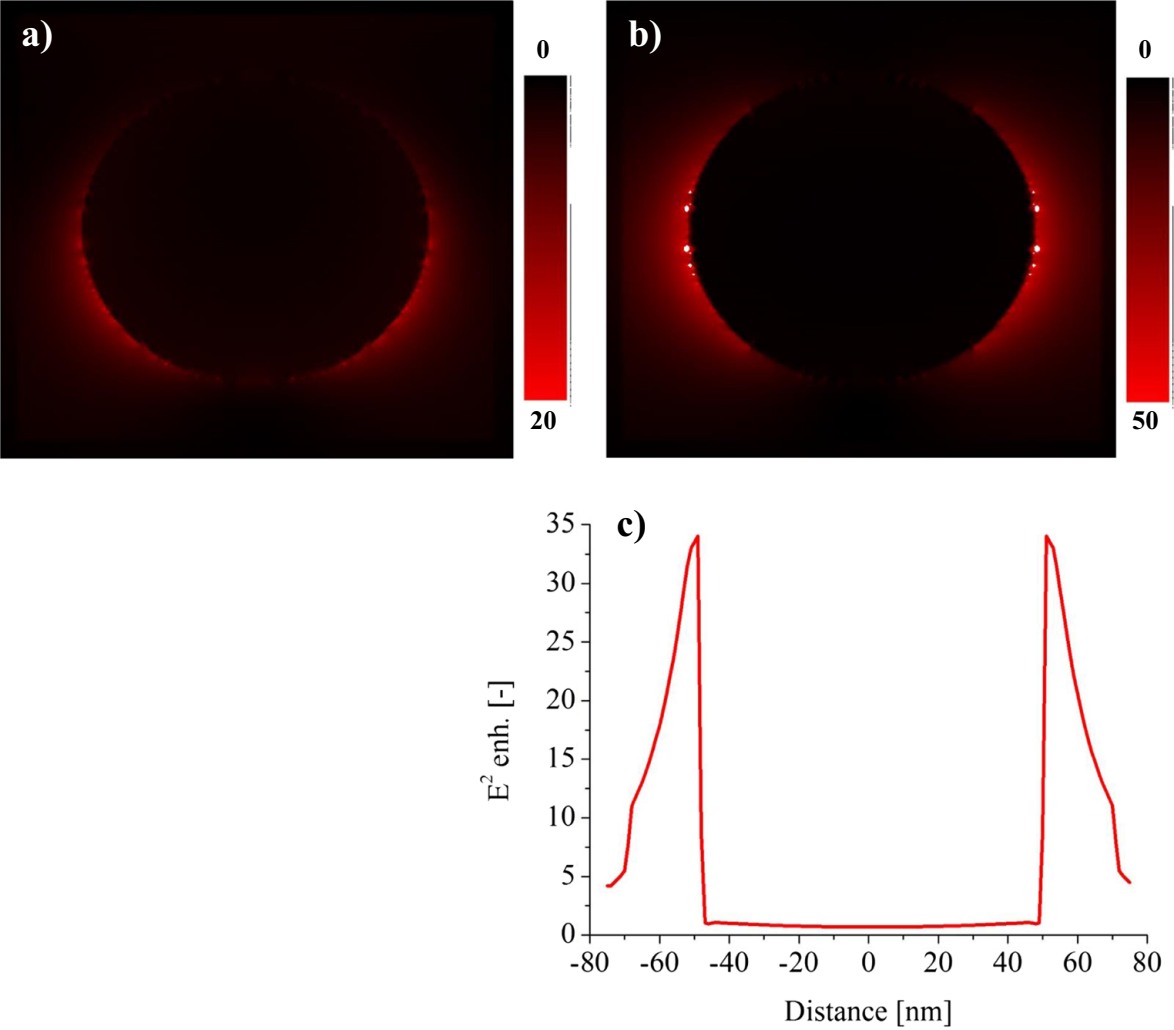
The map of the E^2^ distribution for the 100-nm gold nanoparticle, excited by 514 (**a**) and 633 nm (**b**) with the field profile (**c**)

The electric field enhancement is much weaker for the gold spheres (Fig. 2) when compared with the silver spheres, which is not surprising based on the information presented in the previous paragraph. From the lasers, the most commonly used for the Raman, the silver nanoparticles interact resonantly with the short wavelength excitation lasers like 488 or 514 nm. For these lasers, the electric field energy (E^2^) enhancement can reach the factor of 57.5 and 45, respectively. For the gold NP, the resonant character matches the 633-nm laser when their sizes approach 100 nm. However, the expected E^2^ enhancement is not higher than 34 times. The both types of metals in the spherical form and 20–100 nm size range are almost inactive when the excitation light has the longer wavelength: red (*λ*_exc_ = 780) and infrared radiation (*λ*_exc_ = 830 nm). The E field enhancement is strongly dictated by the match of the excitation laser wavelength and the resonance wavelength. In most cases, the resonant interaction concerns the dipolar plasmonic mode, for which the strongest electric field is located on the equator of the nanoparticle (Fig. 3b). For the larger particles, the driving electric field is not more evenly distributed over the whole NP, resulting in the higher order oscillation modes, like quadrupolar or even hexapolar modes, especially for shorter wavelengths. On the maps of the electric field enhancement, this is displayed as the field distortion (Fig. 3a). Usually, the higher order modest is less intensive, absorption dominates over the scattering, and lower field enhancement can be expected.

In Fig. 3b, some bright spots can be seen, which are out of the color scale shown nearby. These are the artifacts resulting from the imperfections in the simulation method. Namely, the whole model is approximated by the simulation mesh and the averaging of the refractive index within the unit cell may cause the different resonance conditions. This can lead to the artificial field enhancement. This problem can be overcome by refining the simulation mesh; however, this has an enormous impact on the simulation time. In case of the simulations presented in this paper, the reasonable subnanometer sizes of the unit were chosen, and they were confirmed by the preliminary simulations. The simulation mesh was 0.2–0.8 nm linearly for particles sizes from 20 to 100 nm. Additionally, all of the field maps were manually processed and the correct filed profiles were chosen. The data for the field enhancement plots were taken as the maximum values from the field profiles (Fig. 3c). As it can be seen, the highest field enhancement is located near the surface of the plasmonic particles and decays rapidly with the distance. The sharp drop on the field profile at both ends is also artificial and is connected with the presence of the light source box, namely, the TFSF, which allows only the scattered field component to come out. The spectral position of highest field enhancement is not directly overlapping with the maximum on extinction efficiency plot, but usually is shifted to approximately 20 nm towards the longer wavelengths. Generally, in the presented paper, the main attention was put on the comparison of the approximated plasmonic properties of number of the nanoparticles, namely, the resonance position and the electric field enhancement that might be useful for Raman studies of bionanocomplexes.

For the light excitation source with the wavelength of *λ*_exc_ = 633 nm, the enhancement is higher for the gold nanoparticle with the diameter near 100 nm. It is confirmed by the experimental results presented by S. Hong and X. Li [24]. They investigated experimental enhancement factor, as a function of the size of gold nanoparticles in three cases: when the number of the gold NPs was the same, when the surface area of the gold NPs was the same, and when the concentration of gold was the same. The results of the first experiment are consistent with the ones presented in Fig. 2d theoretical calculations for *λ*_exc_ = 633 nm. The experimental data show that the enhancement factor is on the highest level for the largest investigated gold NPs (80 nm). However, when the same concentration of gold is considered, the gold NPs of around 50-nm diameter are the most effective. Similar results were presented, in other papers, where the maximum of the SERS enhancement was obtained for 42-nm AuNPs (at similar *λ*_exc_ = 780 nm) [25] and for 53-nm AuNPs at *λ*_exc_ = 633 nm [26]. When the same NP surface area of gold concentration is considered, the higher concentration of the NPs with the size of 40–50 nm give better SERS signal, than in the case of the nanoparticles of larger diameter (about 80–100 nm) [26]. This might be because there are about 8 times less large NPs, while the field of the enhancement of the smaller ones is only about 2–3 times lower.

From the comparison of the AgNP and the AuNP, it can be seen that gold can support much lower SERS efficiency and additionally, it has a relatively static resonance in case of the wavelength. However, the advantages of this material (e.g., the high stability, the chemical resistance, and the good ability to link molecules adsorption) persuade to find other ways to improve the gold-based plasmonic nanoparticles. In the following part of the paper, some other compositions of the NPs maintaining the gold outer surface will be investigated. It should be claimed that there are other approaches to the change of the plasmonic properties of the metal-based nanoparticles by replacing the simple geometries of the NPs. It is possible to obtain a very high field enhancement, but this requires the much more advanced nanoparticles concepts like the spiky, [27], the nanoporous [28], and the interparticle gap [29] nanomaterials.

**Fig. 4:**
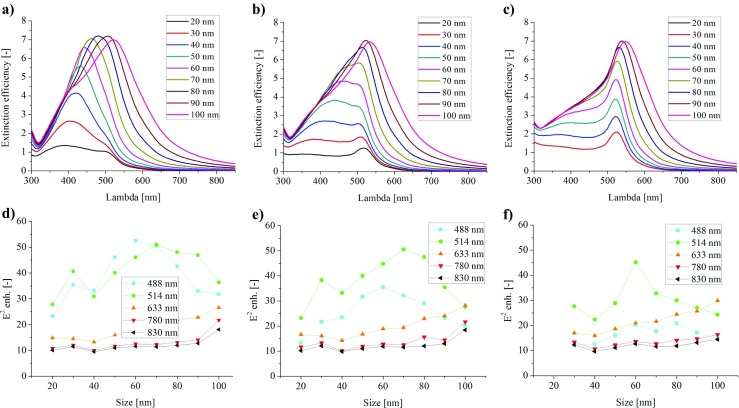
The extinction efficiency and the E^2^ maximal enhancement for the Ag–Au core-shell nanoparticles: **a**, **d** 2.5 nm of Au shell; **b**, **e** 5 nm of Au shell; **c**, **f** 10 nm of Au shell

For that matter, the Ag–Au core-shell structure was investigated. The size of the nanoparticles was the same as for the homogeneous metallic nanoparticles (from 20 to 100 nm). The thicknesses of the gold shell were taken at the account of 2.5, 5, and 10 nm. Within this narrow thickness range, the properties of the plasmonic NPs change from the silver dominated to the gold dominated. For the thinner shells, 2.5 and 5 nm (Fig. 4 a, b), the relatively strong red shift of the plasmonic activity with the increasing particles size can be seen, which is characteristic of silver. Additionally, for small nanoparticles, the two different peaks emerged on the extinction plots. They come from the two metals. For certain size (70–80 nm) the peaks start to overlap, due the stronger plasmon red shift for silver than for gold. For the 10-nm gold shell, this feature on the extinction plot is still present, but less evident (Fig. 4c), even though the maximal extinction efficiency is not higher than for the pure gold.

However, the field profiles for the short wavelengths of the excitation laser have been significantly changed (Fig. 4d–f). For the 2.5 nm of Au shell, the two maxima of the E^2^ enhancement can be expected, when using the 488 and 514-nm laser excitation wavelengths and each of them should support over 50-fold of the enhancement for the 60–70-nm particles. It is even higher enhancement than it was predicted for the pure silver. When the shell thickness increases to 5 nm, the strength of the resonance excited by 488-nm light source decreases, but it remains still high for the *λ*_exc_ = 514 nm. The enhancement factor is also high for this excitation wavelength of 10 nm of Au shell and the size of the NPs about 60 nm. The overlapping contributions from the two metals can be expected for the shorter wavelengths, but beyond the Au dipolar resonance (*λ*_exc_ of 633 nm or longer), the expected field enhancement is not better and almost the same as for the pure gold NPs.

This strong interaction with the 514-nm laser is important from the experimental point of view, if the synthesis of the nanometer precision of the shell thickness would be challenging. For other excitation lasers, no additional benefits are predicted from the Ag–Au core-shell structure. Concluding this part, we have shown that by keeping the gold outer surface, it is possible to tune the resonance to the short excitation wavelengths (488 and 514 nm) and at the same time to take the advantage from the higher field enhancement. Then, the question remains how to tune the resonance to the longer excitation wavelength.

The nanorod geometry offers a good resonance tunability. In the nanorod, the dipolar resonant mode splits into the two modes: the longitudinal and the transverse mode [12]. By increasing the length of the nanorod resonance, it can be easily redshifted. The properties of the Au nanorods having the fixed short diameter of 70 nm and the length varying from 55 to 155 nm were studied. The curvature on each side was fixed to 20 nm.

**Fig. 5:**
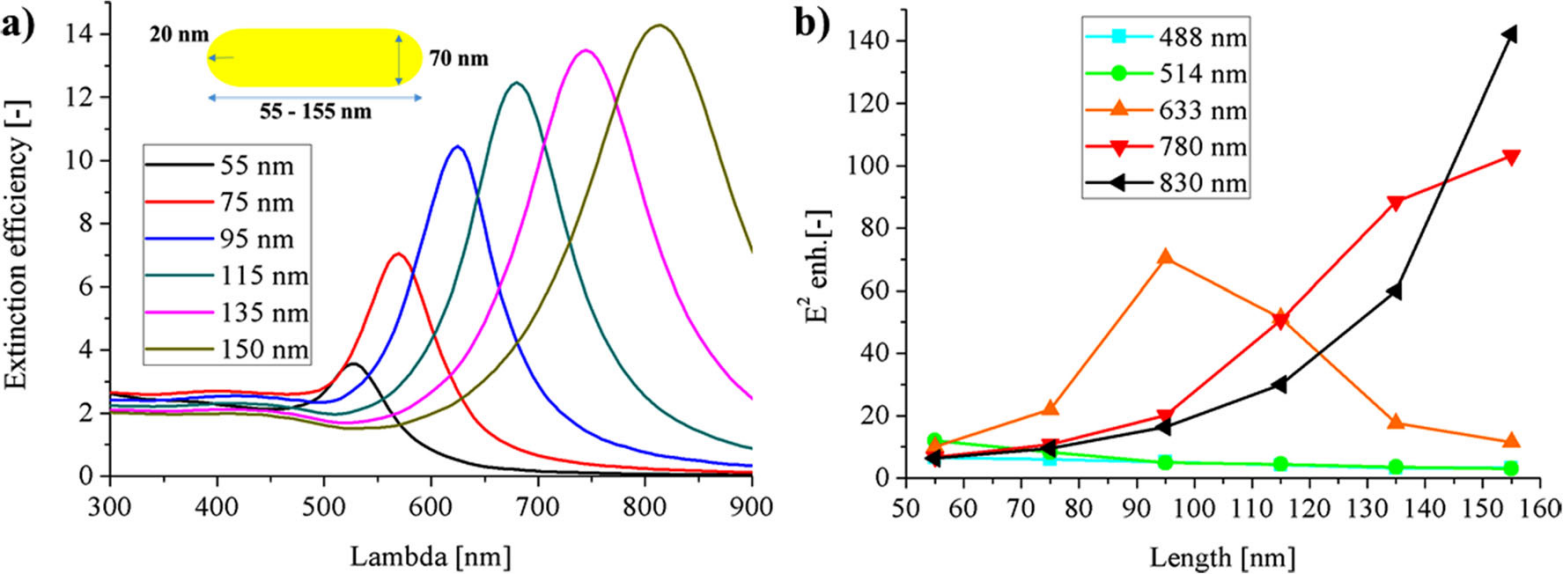
**a** The extinction efficiency and **b** the E^2^ maximal enhancement for gold nanorod

The extinction efficiency of the longitudinal modes in the gold nanorods caused by the light polarized along the long axis of the nanorod is shown in Fig. 5a. It can be seen that the resonance strength increases significantly with the nanorod length. For the nanorod of the 155-nm length, the extinction efficiency may reach approximately 14. For the gold nanorods of such length, the resonance can be easily tuned to the infrared region. Therefore, with the increasing length, we predicted that the long wavelength excitation lasers fitted well to the resonance conditions. For the longitudinal mode, the E field enhancement takes place exclusively at the ends. In the field analysis, the very high enhancement factor was found, which was attainable at the ends of nanorod: ~ 80 for *L* = 95 nm and *λ*_exc_ = 633 nm, ~ 100 for *L* = 155 nm and *λ*_exc_ = 780 nm, and ~ 140 for *L* = 155 nm and *λ*_exc_ = 830 nm. The E^2^ enhancement is almost 3 times higher than predicted for the silver nanosphere and 5 times than the gold one in their respective optimal configurations (Fig. 2c, d). This enhancement can be even higher. When considering the decrease of the short dimension, while keeping the length constant, the resulting field enhancement will be higher, due to the increased curvature. However, the strong field enhancement is not the only aspect of the potential SERS measurements of the bionanocomplexes. When other aspects are taken into the account, one can specify some drawbacks. Firstly, the nanorods will interact in this way, only with the one light polarization; the second polarization of such long wavelength will cause a very weak transverse interaction far beyond the resonance conditions. Thus, only half of the laser intensity will be used. Moreover, in the colloidal suspension, the NPs are in the constant rotating motion around three axis and the properties of the investigated configuration will be hardly attainable or at least very blurred. Secondly, in order to gain from the field enhancement at the specific spots, the particles have to be equally covered throughout the whole surface area by the investigated molecules, enzyme etc. With the increasing length of the rod, automatically higher surface area has to be covered by the molecules, which will hardly contribute to the signal. Thus, the higher concentration of the investigated molecules is necessary. Finally, the gold consumption for it will be increased.

The answer to the mentioned above drawbacks can be again the spherical core-shell structures, but this time with the dielectric core. They are fully symmetric and each part of the surface can be found in the hotspot, additionally, have lowest surface to the volume ratio and the aspect of the amount of gold per particles is much more favorable in case of the core-shell geometry. The last one may have a great importance for the real application for fast identification without the possibility to be reused. As a dielectric core, we considered the two common materials, the silica and the titania with the refractive index of 1.46 and 2.26 respectively. Since the metal–dielectric interface has a high surface energy [30], it could be harder to experimentally obtain the shell thickness of the several nanometers. Therefore, we decided to study the 10-nm thick shell.

**Fig. 6:**
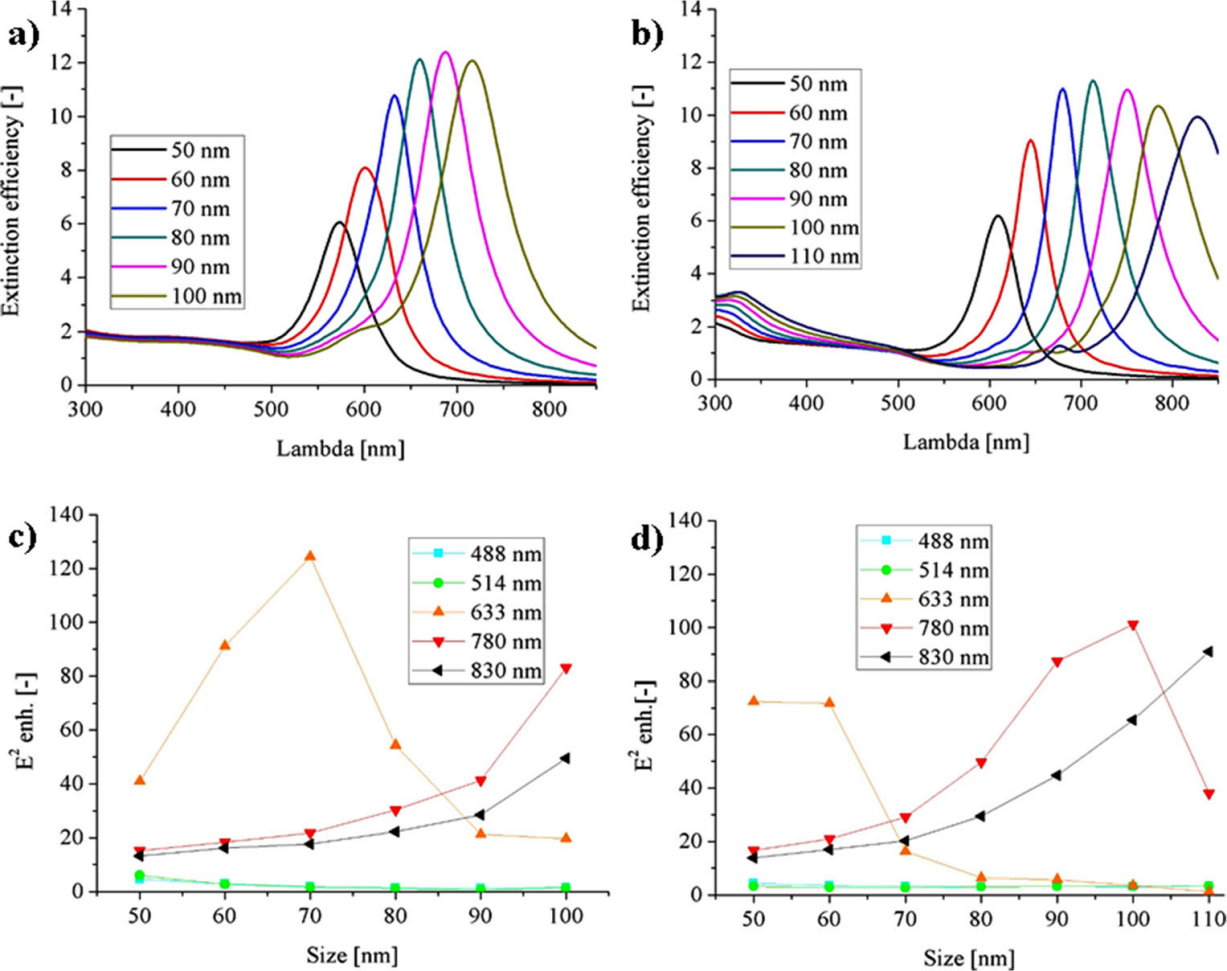
The extinction efficiency and the E^2^ maximal enhancement for SiO_2_–Au (**a**), (**c**) and TiO_2_–Au: (**b**), (**d**) core-shell nanoparticles

It can be seen from Fig. 6 that the resonance is efficiently red shifted to the edge of the visible range for the larger particles with the silica core and shifted even further into the infrared for titania core, due to its higher refractive index. The extinction efficiency is high—between 10 and 12 for the optimal size. From the field analysis, it is found that depending on the size of the NPs, one can gain the three different wavelengths *λ*_exc_ = 633, 780, and 830 nm. The field enhancement is much higher than for the simple gold sphere and it approaches the values for the optimal case of the nanorods from the previous analysis. It can be around 124 times for the SiO_2_ and *λ*_exc_ = 633 nm, 101 times for the TiO_2_ and *λ*_exc_ = 780 nm, and finally 91 times for the TiO_2_ and *λ*_exc_ = 830 nm. It has to be mentioned that the actual maximal values can be located at some intermediate nanoparticle sizes. The one possible drawback of the dielectric core of the NPs metallic shell is the increased Raman spectrum of the dielectric material, which can act as the increased background for the investigated molecule spectrum. It is because the E field localization partially takes place in the dielectric core as well.

## Summary

The simulation of the plasmonic effects caused by the silver (AgNPs) and the gold nanoparticles (AuNPs) have been performed. The special attention was put on the AuNPs, which are especially useful for the SERS applications for the bionano-carriers (the proteins, the enzymes, and the nucleic acids) analysis. We studied the possibility of the effective tuning of the plasmonic resonance of gold nanoparticles on the different wavelengths, using the simple geometries, even though gold is known for the relatively static resonance wavelengths. It was shown that the resonance of the nanoparticles having gold as the outer surface can be adjusted to the excitation laser of the most commonly used for the Raman investigations, e.g., with the wavelengths from 488 to 830 nm adjusting their structure and diameter. Particular emphasis was placed on the core-shell structures, predicting their high potential. The electric filed enhancement that would contribute to the SERS factor of the Raman investigation of the bionanocomplexes was also predicted by the performed simulations enabling to compare the nanoparticles between themselves.
